# Farewell Ceremonies: How Older People Practice Death and Bereavement

**DOI:** 10.1007/s10746-024-09767-w

**Published:** 2024-11-28

**Authors:** Hans-Georg Eilenberger, Marjolein de Boer

**Affiliations:** 1 University of Humanistic Studies, Utrecht, The Netherlands; 2 Tilburg School of Humanities and Digital Sciences, Department of Culture Studies, Tilburg University, Tilburg, Netherlands

**Keywords:** Ageing, Existentialism, Simone de Beauvoir, Bereavement, Mortality

## Abstract

Later life is often seen as a time of losses. Through the death of loved ones and the dwindling of bodily capacities, older people are increasingly confronted with their own mortality. As losses accrue across different domains, they form a unique existential vantage point. We aim to shed light on this understudied dimension of later life by analysing older people’s everyday practices of sense-making. Drawing on the findings of a qualitative interview study (*n*=16, aged 65-93), we identify three distinct practices by which older people make sense of death and bereavement: timing, communing, and missing. We conceptualise these practices as “farewell ceremonies,” a term we borrow from Simone de Beauvoir. The “farewell ceremony” describes a period of incremental goodbyes, which characterised the last ten years of Jean-Paul Sartre as well as the death process of Beauvoir’s mother Françoise. Beauvoir captures these farewell ceremonies in her memoirs *Adieux: A Farewell to Sartre* and *A Very Easy Death*, which frame our philosophical reflection on practices of timing, communing, and missing. Bringing together Beauvoir’s literary and philosophical work with our empirical findings, we propose an integrated view on death and bereavement in later life that centres the intertwining perspectives of self and other.

## Introduction

The older you get the more people fall ill, pass away. It’s someone every week… nearby or further away. That’s just the way it is, that’s just the way it *is*. (Arie, aged 71).

This statement by our interview participant Arie reflects the common association of later life with illness, bereavement, and loss. There is a rich literature on the effects of bereavement on older people’s health, social relations, and life situation (e.g., Holm et al., 2019; Infurna & Mayer, 2019; Kersting et al., 2011; Tornstam, 2005). The death of loved ones is one very prominent source of grief in later life. Other sources include the loss of health, bodily functions, independence and social roles. These losses tend to accumulate over a lifetime; they form a “pool of grief” (Moss & Moss, 2003: 5) and may shift one’s existential outlook on life in ways that are as yet poorly understood (Fang & Carr, 2024; Lekalakala-Mokgele, 2018). In confronting the death of others as well as their own mortality, older people use diverse practices of sense-making. These practices require (scholarly) care and attention, especially because of the ambiguity and immense diversity of experiences. Our paper addresses this concern by examining older people’s dealings with death and mortality. It is based on a series of in-depth interviews with older people, which we interpret through the lens of existential-phenomenological philosophy, and especially through the works of Simone de Beauvoir.

Drawing on Beauvoir’s philosophy, our paper illuminates aspects of older people’s existential situation that are often overlooked in the psychological and gerontological literature. Psychological studies on bereavement suggest that the majority of older people are fairly resilient and able to make sense of their loss. At the same time, they may face more difficulties in doing so than their younger peers (Infurna & Mayer, 2019; Kersting et al., 2011; Neimeyer & Holland, 2015; Newson et al., 2011). There are indications that pathological patterns of grief, referred to as “prolonged grief disorder” or “complicated grief,” are more prevalent among older people (Kersting et al., 2011; Newson et al., 2011). While these findings certainly have their use, they only reflect isolated and quantified aspects of the total existential situation of older people. The same pattern is apparent in studies on death anxiety among older people (Fortner & Neimeyer, 1999; Ma-Kellams et al., 2021). Gerontological frameworks such as gerotranscendence give more space to the spiritual aspect of growing older; yet, they merely treat death as an object of fear and do not address the significance of the other’s death (Tornstam, 2005).

To address these gaps in the psychological and gerontological literature, we turn to existentialist-phenomenological approaches to illness and health (Abrams & Setchell, 2018; Leder, 2018). Existential phenomenology explores how everyday lived experience discloses a person’s basic orientation toward the world; it centres the meaning of the experience rather than the objective factors that underlie it. Our aim is to provide a deeper understanding of the existential dimension of later life based on an analysis of people’s practical dealings with death and loss. We describe practices of timing, communing, and missing, which we derive from a series of in-depth, semi-structured interviews with older people (*n* = 16; aged 65–93). These practices are conceptualised as “farewell ceremonies,” a notion that we borrow from Simone de Beauvoir’s philosophy of existence. In contrast to other prominent figures in the phenomenological tradition, such as Martin Heidegger, Beauvoir (1996) studied mortality and bereavement in the context of later life. Her philosophical work pivots on the notion of situated freedom, suggesting that existing involves assuming one’s situation by means of actions and choices. Her memoirs of the deaths of her mother Françoise (Beauvoir, 1965) and of her partner Jean-Paul Sartre (Beauvoir, 1984) illuminate how the notion of situated freedom functions in the context of bereavement: as a farewell ceremony. While Beauvoir herself has not worked out this notion in depth, we will conceptualise it further on the basis of our empirical material.

## Beauvoir’s Farewell Ceremony

Since her early years, Simone de Beauvoir harboured a fear of death. As she states in her memoir *The Prime of Life*: “From the time I knew I was mortal I found the idea of death terrifying” (Beauvoir, 1992: 474). Beauvoir’s preoccupation with death and mortality marks much of her philosophy, fiction, and memoirs; it grew in intensity as she advanced in age, culminating in the two (auto)biographical works *A Very Easy Death* (Beauvoir, 1965), which describes the death of Beauvoir’s mother Françoise from stomach cancer, and *Adieux: A Farewell to Sartre* (Beauvoir, 1984), which narrates the last ten years of Jean-Paul Sartre.^1^

The philosophical implications of *A Very Easy Death* and *Adieux* emerge most vividly when considering the content of these works alongside their form. Narrating the deaths of her mother and partner, Beauvoir not so much theorises mourning as she performs it. It is in the literary presentation and the stylistic choices that Beauvoir’s (auto)biographies convey a conceptual insight into the nature and workings of mourning. The notion of the “farewell ceremony” (*cérémonie des adieux*) succinctly captures what is at stake in these “literary tombs” that Beauvoir erects for intimate others. The term originated from an ironic exchange between Beauvoir and Sartre:

This time we had lunch together at *La Couple*, where Sylvie [Beauvoir’s adopted daughter] was to come for me at four o’clock. I stood up three minutes before the hour. He [Sartre] gave me an indefinable smile and said, “So this is the farewell ceremony!” I touched his shoulder without replying. The smile and the words stayed with me for a great while. I gave the word farewell the ultimate meaning it was to have some years later; but when that happened I was the only one to say it. (Beauvoir, 1984: n.p.)

In Beauvoir’s narration, her everyday goodbye from Sartre foreshadows their ultimate separation nine years later. The passage marks a central moment in *Adieux* where the timelines of the present and the future come to intersect. Although Sartre is presently alive, the scene opens a window to his future absence, his death. The notion of the farewell ceremony contains this sense of anticipatory grief as well as the reversal of roles between Sartre and Beauvoir: it starts with Sartre bidding farewell and ends with Beauvoir, the mourner, saying the words alone. More than denoting a single experience, the farewell ceremony stands in for the entirety of Beauvoir’s and Sartre’s last years together—“the farewell ceremony” (*La cérémonie des adieux*) is, after all, the title of the original French memoir. It characterises a period of repeated, incremental goodbyes completed by Beauvoir’s act of writing. As in *A Very Easy Death*, Beauvoir’s writing transforms her experience of the other’s death into a testimony of what it means to be mortal together. To appreciate the philosophical weight of this transformation requires an examination of Beauvoir’s conception of freedom and intersubjectivity.

The idea of the farewell ceremony reflects Beauvoir’s understanding of human existence in terms of situated freedom. In a basic ontological sense, freedom is the capacity to take a stance vis-à-vis one’s situation. This ontological freedom acquires a specifically ethical sense in Beauvoir’s work, which emphasises the “doings” of an agent in contrast to their “being” (Beauvoir, 1976). Human beings exercise their freedom by giving meaning to the events they undergo in light of their projects, thereby placing themselves in a continuous process of becoming. In *The Ethics of Ambiguity*, Beauvoir (1976) identifies internal and external limits to the use of one’s freedom, which she links to the fundamental ambiguity of human existence: the fact that human beings simultaneously constitute their reality and are constituted by it (see Kruks, 1990: 91–99). Death makes this ambiguity manifest. On the one hand, it cuts short human freedom in an obvious way; on the other hand, it is only within the limits of a finite life that choices become meaningful (La Caze, 2004–2005). Beauvoir wavers between these two possibilities in her writings on death, which includes *Adieux*. The notion of the farewell ceremony, which emerges from this work, encapsulates Beauvoir’s philosophical and existential struggle with death. It shows how the death of a loved one annihilates meaning but at the same time acquires ever new significations through the choices of the mourner, their acts of witnessing, writing, and burying.

Acts of mourning make sense of loss and death and re-affirm the co-existence of mourner and deceased. In Beauvoir’s philosophical vision, death is an intersubjective phenomenon, but ambiguously so. While death ultimately separates the mourner from the deceased, it also signifies their common participation in the generality of life. If everybody is mortal, if everybody faces the dread of annihilation, there is space for recognition and solidarity. *A Very Easy Death* captures this vision like few others of Beauvoir’s writings, as it describes in great detail the fears, vivacity, and suffering of Françoise de Beauvoir in the run-up to her death. The memoir reports the rapprochement and the rekindling of an affectionate relationship between mother and daughter. Identifying with her dying parent, Beauvoir revolts in her name against the “unjustifiable violation” that is death (Beauvoir, 1965: 106). This blurring of perspectives co-exists with the recognition of an unbridgeable difference between mother and daughter, self and other. Beauvoir navigates the tension between difference and identification through the farewell ceremony of writing. It is writing that allows her to take a defiant stance against the death of the other, to shout “the final ‘No’ for her mother and for herself” (Holveck, 1989: 78).

Although Beauvoir did not systematically work out the notion of the farewell ceremony and did not explicitly position and discuss it in the context of existentialist philosophy, it arguably suggests a philosophical vision of death and mourning that is at odds with the orthodox Heideggerian position. Martin Heidegger (1962) produced the classic phenomenological analysis of death, famously describing human existence as “being towards death.” For him, death proper is a horizon of finitude that orientates human beings toward the fundamental question of what it means to be. In conceptualizing death, however, Heidegger largely disregards the empirical context of somebody’s death and treats the death of the other as existentially insignificant. Bereavement, he maintains, is no proxy for the significance of death proper, which “by its very essence, […] is in every case mine, in so far as it ‘is’ at all” (Heidegger, 1962: 284). This disregard for the other’s death has been the subject of critique (Dastur, 1996; Heinämaa, 2010a)—a critique that resonates with Beauvoir’s approach to death.

Beauvoir’s notion of the farewell ceremony is acutely attuned to the existential situation of later life, with its shared embodied sense of finitude. For her, death is neither solipsistic nor static, but frequently disclosed through practical engagements and shared with others. As a practice of *situated* freedom in the face of loss and death, the farewell ceremony is indeed deeply contextual. Beauvoir recognises that attitudes toward death typically change in the course of a person’s life (La Caze, 2004–2005: 146). In *The Coming of Age*, crucially, Beauvoir (1996) describes the slow diminishment of one’s subjectivity that accompanies the deaths of loved ones in later life. Advanced age presents existential possibilities of mourning, such as farewell ceremonies, which we will discuss in the following analysis.

## Methodology

To explore the farewell ceremonies of older people, the first author conducted in-depth semi-structured interviews with sixteen older people in two large cities in the Netherlands. The ages of participants ranged from 65 to 93 years, with the majority being younger than 74. The sample included nine women and seven men. Their educational and family backgrounds were varied. Five of them had both children and romantic partners, five had either children or partners, and six were childless and without a partner. To safeguard the privacy of participants, their data were anonymised and pseudonyms were used throughout this paper.

The sampling started once the ethics review board of the School of Humanities and Digital Sciences at Tilburg University had approved the research proposal. Participants were alerted about the interview study via a number of channels: an advertisement in the newsletter of a local community centre, a sports club, a Whatsapp group, a meeting group for queer older people, and through the network of a community nurse. Two participants learned about the study via another participant. To be included in the sample, people had to be at least 65 years old and live independently. This age threshold reflects conventional definitions of later adulthood (see APA, n.d.). Another consideration in the sampling process was a high diversity in terms of ages, genders, and sexual orientations. The fact that most participants belong to the “young-old” (aged between 65 and 74 years) reflects the prevalence of this group in the targeted organisations.

The first author conducted the interviews at the participants’ homes or on the campus of Tilburg University. He used a topic list informed by the key phenomenological categories of temporality, spatiality, embodiment, and intersubjectivity (Van Manen, 1990). Interview questions concerned an old photo that participants were asked to bring (temporality),^2^ their thoughts and feelings about ageing (embodiment), places they frequent daily (spatiality), people who care for them (intersubjectivity) as well as questions about the (in)significance of the COVID-19 pandemic for them. Interviews lasted between one hour and twenty minutes and three hours. The first author made sure that there were sufficient breaks and that participants had ample time to navigate emotionally challenging topics. He also reminded them of the possibility to skip questions, or to terminate the interview without giving a reason. Having conducted the interviews, he sent brief summaries to the participants and used that occasion to check in with them. The interviews were recorded, transcribed verbatim, and pseudonymised upon transcription. In this paper, we use English translations of the original Dutch statements.

Data analysis followed the framework of Reflexive Thematic Analysis (Braun & Clarke, 2006, 2013). In the first step, the first author applied open codes to the material, such as “what corona does to you,” “defining memories of the deceased,” and “my/your/our dying process.” He then grouped the codes into 22 sense-making practices. These practices included “inhabiting an ending world,” “death cleaning,“ and “acting out of bounds.” Keeping in mind the phenomenological categories of temporality, intersubjectivity, and otherness, the first author, with assistance and feedback from the second author, clustered the different sense-making practices into three overarching themes: *timing*, *communing*, and *missing*. This clustering involved a back-and-forth movement between different levels of analysis; the first author searched for common meanings among the sense-making practices while continuously relating them to the phenomenological framework. He wrote the paper, incorporating the feedback of the second author, who also contributed to the writing process.

## Findings: Three Kinds of Farewell Ceremonies

This section explores the farewell ceremonies that we gleaned from the stories of participants. By “ceremonies” we do not mean formal rituals, but all kinds of activities that make sense of death and bereavement. They are everyday doings that may appear mundane or even trivial, as reflected in Beauvoir’s (1984) writing on her protracted farewell from Sartre. Farewell ceremonies are performative: they offer ways of dealing with and making sense of loss and death, and of coming to terms with otherness. Relating episodes of bereavement and other losses, participants frequently conveyed a sense of the unfamiliar, strange, or unexpected. Participants’ accounts show how bereavement, loss, and death upset the habitual distinction between self and other: the difference between my body and the other’s body, between my death and the other’s dying. It is for this reason that we decided to treat the perspectives of self and other together and explore their intersections. The following subsections are each dedicated to a different farewell ceremony: practices of timing, communing, and missing, which thematise the participants’ ways of sense-making in relation to their own death, their communities, and the dead or dying other.

## Practices of Timing: The Mortal Self

In *The Coming of Age*, Beauvoir (1996: 361) argues that age radically changes one’s relationship to time. Living in the awareness of death, older people experience the future no longer as a limitless expanse but as narrow and bounded. This shortening of the future horizon upends the narrative structure on which the possession of the past relies. Losing the ability to animate their past in future-oriented projects, older people may find their memories frozen and without temporal anchors (Heinämaa, 2014: 180). As our study shows, the prospect of death plays a complex role in participants’ experience of time. To them, death is not a static limit but a constitutive element of experience. Facing death, participants recalled past events, prepared for an uncertain future, and highlighted their co-presence with others. These ways of relating to death create a temporal frame for the experience of ageing in later life. We refer to them as practices of timing.

The following quotes show practices of timing in various everyday social encounters. In the comparison with peers, some participants develop a sense of temporal order or hierarchy, wondering when it would be *their turn* to die.

But I think I—yes, I see that in other friends who are also around that age. They’re all obviously facing that same thing. And certainly when you drink tea with them, you think—the question is: who goes first? Things like that, that all plays into it. (Ico, 72)

That sometimes… that is, yes, tricky, because that’s where you get anxious. But yes, then you think: yes, one day it will be my turn… (Arie, 71).

Both Ico and Arie appeal to the metaphor of turn-taking. Like in a game or ritual, there is a temporal order of things: somebody has to die first, then another will follow, then another… This way of timing death emerges from concrete social activities, such as drinking tea with friends. It suggests a mortal self that is part of a larger time scale involving other people. In this sense, the otherness of the mortal self is mediated by the presence (and expected absence) of others. In the following quote, the mediating role of others appears in an even more tangible way:

No, I have to tell you honestly—I told that to my medium as well: there were nights when I have not been able to sleep. I got a lot of fear of death now. While I know that I have cancer—that sits, that stays in your body, never goes away, you stay tired. And I sleep very badly lately. (Hugo, 69)

Hugo addresses his growing fear of death by seeking spiritual advice from a medium, who literally mediates between his present life and his expectations for the future. In conversation with his medium, Hugo is able to voice his deepest anxieties about the return of his earlier cancer and the prospect of death itself. We understand this as a practice of timing death, a particular way of looking at death through the lens of fear. In Hugo’s statement, fear is associated with the unpredictable materiality of the body: its potential for cancerous growth, exhaustion, and sleeplessness. The body appears as the other of the mortal self, the other who comes to view through particular practices of fearing. A similar dynamic is at play in the following quotes:

No, no. But should I not be able to do anything at all anymore… that I cannot walk or talk or hear at all, they may give me an injection [i.e., euthanise me]. (Nienke, 69)

I hope for an, um, peaceful death, but… I hope that however it goes, I realise now, that at least, yes, then my family, so to speak, can be there in that sense to make decisions so that I don’t lie somewhere croaking for a long time or whatever. (Eveline, 65)

Nienke and Eveline time their deaths by making arrangements for a good, dignified ending. Their references to euthanasia and living wills, respectively, reflect the wide availability (and social acceptability) of these instruments in the Netherlands. Focusing on the practicalities of avoiding suffering and indignity allows them to re-present death in a measured, less frightful manner. Fear is contained by a sense of self-efficacy and, in Eveline’s case, trust in her family. In the background, however, fear is still looming. Like Hugo, Nienke and Eveline fear the unpredictable deterioration, the otherness of the dying body. In contrast to these instances of “mortal fear,” the following quote combines the contemplation of death with a sense of gratitude:

Because we really have with much pleasure and with everything, have really enjoyed, um, everything. We got free, we had from the railways, free travel. And we really took advantage of that a lot during the holidays. And, um, yes, we really, um, we saw an awful lot, we travelled a lot. And then at some point you have to be satisfied. (Lisbeth, 87)

Activities play a major role in Lisbeth’s account. She recalls the travels together with her husband and draws on these happy memories as she comes to terms with their nearing deaths. In Lisbeth’s case, timing death involves remembering her life in a spirit of gratitude. It brings the goodness of the past to bear on the prospect of an ever-shortening future. This resourceful assumption of death comes with an imperative: “you have to be satisfied”. The “you” in this statement could be a manner of speech, but it might also reflect an ongoing dialogue with Lisbeth’s husband, whose age-related discontents were a reoccurring theme in the interview.

The last set of quotes explicitly relates the experience of time to the concrete nearness of death. Reflecting on his current life circumstances, Evert describes an atmosphere of loss that surrounds him and narrows his perspective on the future:

I also get surrounded by illness, death now. My brother is in palliative care, he is dying. But there are many people, uh, around me who have something or have already died. (Evert, 66)

And also, when I think about that now: the essential difference of… that… that… that carefree and… that very broad perspective towards the future that you—at least I had then—that is no longer there. (Evert, 66)

Evert practices “timing” not by actively projecting himself into the world but through the passive apprehension of his finitude. He feels *surrounded* by illness and death, a formulation that conveys a sense of constriction and powerlessness. This being said, Evert’s practice of timing clearly involves an act of identification with the suffering of others, such as his terminally ill brother. The illness and death of people around him reflect the deterioration of possibilities that Evert associates with later life. This collapse of life’s possibilities also has ramifications for Evert’s experience of time. Comparing his present attitude toward life with his perspective twenty years ago, he observes a remarkable shift in his sense of the future: what used to feel like an open horizon has turned into a narrow encirclement.

Evert’s case reveals the connection between temporal and intersubjective experience that is implicitly present in other participants as well. The practices discussed above—drinking tea with friends, conferring with a medium, arranging for a dignified death, and recounting past travels—all imply a co-experiencing other in some way or another. In addition, they all disclose particular modes of relating to the end of life: hopefully, pragmatically, apprehensively, anxiously, etc. Practices of timing not only integrate the prospect of death into the temporal flow of experience; they also reflect intersubjective and social dynamics. This finding is in line with Beauvoir’s (1996) thesis that temporal experience changes with age. The nearness of death reconfigures a person’s lived experience of time, but this nearness should not be construed in individualistic terms. As Beauvoir (1996: 366) powerfully shows, subjectivity is sustained by living relationships with loved ones and shattered by their loss: “The death of a friend, of one who was close to us, not only deprives us of a presence but of the whole of that part of our lives that was committed to them.” Farewell ceremonies of timing reflect this intrinsic connection between the mortality of the self and the mortality of the other.

## Practices of Communing: The New “We”

Beauvoir (1984) dedicates *Adieux* “[t]o those who loved Sartre / who do love him / who will love him”. In this, she indicates that farewell ceremonies are not private activities but are typically orientated toward a social context, a community of mourners. If farewell ceremonies are inherently social, they also aim at the re-creation of sociality. Confronted with repeated bereavement and their own mortality, older people often find their social existence under siege. To counter the weakening of community ties and the shrinking of belonging, participants engaged in practices of communing. These practices include funeral arrangements, dating, caring, and passing on material objects. Communing can take a great variety of forms, but it always centres on the creation of a new sense of “we,” of a subjectivity beyond the individual. This generative aspect of communing is well illustrated by Eveline’s narration of her mother’s funeral:

But then I also noticed the whole week—was almost a week—yes, to be there during such an intense time—and that too—it was very intense. And then particularly with them [other funeral guests]. And we kept trying to contact others who we knew had been important to my mother and who couldn’t go to the funeral to—we went to the crematorium and made arrangements and then they came too. And then we all sat together. And then two of my mother’s neighbours, so to speak, who lived in the same complex. And then… yes, then I’m really in my element. I love being there. Yes, I cherish that. I think: yes, that’s my family. (Eveline, 65)

Eveline’s practice of communing involved contacting the important people in her mother’s life and making sure they are included in the funeral rites. Alongside the emotional intensity of the situation, she highlights the connection she felt with her fellow mourners. Eveline’s choice of words reflects her expanding sense of community: while “we” initially only refers to herself and her brothers, it eventually encompasses all the people who come together at the funeral, Eveline’s newly found “family.” Communing with her fellow mourners in this way, Eveline effectively recreated the network of relationships that her mother had embodied. Her story recalls the notion of “closing the circle,” which came up at another point during the interview. It designates Eveline’s desire to reconnect with her roots, to return to her place of birth after many years of absence.

As the following quote shows, practices of communing not only re-create families but also play an important role in romantic relationships:

And so when I, when [my husband] Daan had passed away, almost a year after that, then all of a sudden [that other man] texted on Whats[app]—I don’t remember—like: how are you doing? And bla bla bla. And: would you like me to come over for coffee? Well yes, come. And he came…… It was instantly *wam*… That was almost four years ago now… but I’m still in love with him. (Arie, 71)

The story of Arie highlights the sudden sensation of falling in love (“wam”) and includes the concrete acts of texting and drinking coffee that led to this rekindling of desire. His statement reflects an overlap between grief, on the one hand, and desire for love and intimacy, on the other hand, that was present throughout the interview. In his pursuit of a new relationship, Arie, too, creates a new sense of “we” in the aftermath of his husband’s death.

If practices of communing are a way of calling out to the other, they also require an appropriate medium and another person who can actually heed the call. We can observe a similar dilemma in relationships of care, as the following two quotes illustrate:

I remember: a good friend who died of cancer a while back, yes, several years back, but we knew that [it] was finite. That as friends, you then say: she was alone, she had a daughter, an adult, who lived abroad. You know what? We set up a schedule, we will take care of her. So, we will make sure she is not alone, we will cook for her, we will look after her, we will keep her company, we will get books from the library for her. I think that’s very important, I think that’s an essential part [of friendship]. (Evert, 66)

But I… I always find it annoying to ask for something. Because also with the boys, then I think: but you always have to wait… People have their own lives too. And [our son], he has his own things. […] So he can’t turn up all the time, he can’t. (Lisbeth, 87)

Evert and Lisbeth find themselves at opposite ends of the care spectrum: while Evert cares for his dying friend, Lisbeth receives care from her family. In response to the increasing vulnerability of his friend, Evert set up a care schedule, kept her company, cooked, and picked up books for her. These practices of communing redefined their friendship, as old ways of togetherness were being lost. By contrast, Lisbeth’s practice of communing consists in waiting. Waiting for care puts her in a dependent relationship with family members who are often away and have their own lives to attend to. As Lisbeth’s wording suggests, she would rather not be part of that carerelationship, that “we,” if she had a choice. Care, it appears, involves different practices of communing, with sometimes contrasting meanings. The same is true in the case of family inheritance, as the following quotes suggest:

It even goes so far—now that I think about it—that my daughter’s daughter wore a hat—who is now two, two-and-a-half—wore a hat that my oldest sister—and she is seventy-six—had worn when she was that young. (Rolien, 67)

My mother [when she passed away] lived alone in a very large house in city for a very long time. And she didn’t want to leave there. And that house had three floors with God knows what stuff, all of it was packed. And, yes, then I thought: well, I don’t want to be the same. […] So I was like: yeah, I don’t want to do that to my children. (Elske, 73)

In their statements, Rolien and Elske discuss the passing on of material objects from one generation to another. Rolien’s story illustrates how an heirloom is created through a practice of communing: the act of passing on that connects grandaunt and grandniece. The intergenerational “we” that emerges in these processes signifies homeliness and belonging. By contrast, Elske’s intergenerational “we” appears burdensome. When Elske’s mother passed away, she left it to her daughters to clear out her cluttered home. This experience, which was repeated when Elske’s sister died, compelled Elske to never impose the same burden on her own children. Her practice of communing, then, is precisely the refusal to pass on a mass of objects, to avoid creating the same intergenerational burden that she had to shoulder.

Participants engaged in practices of communing when confronting the death of a loved one or when faced with their own finitude. Death and finitude upset one’s habitual sense of “we,” that is, one’s ingrained ways of being in the world with others. Practices of communing encompass participants’ responses to the disruption of their social world, leading to the creation of a new “we.” While bereavement research has long recognised the importance of social support as a predictor of psychological outcomes (Logan et al., 2018; Mason et al., 2020), it is largely unclear how mourners themselves interpret social support and which behaviours they consider helpful (Cacciatore, Thieleman, Fretts, & Barnes Jackson, 2021). In contrast to this literature, our findings highlight the ambiguous and non-instrumental nature of “social support.” Communing is not a coping strategy; it does not cast others as supporting actors in the story of one’s own healing. When the “we” is lost or challenged, older people will inevitably create new social worlds, but these worlds may not be harmonious, wholesome, or even desirable. Practices of communing, in this sense, reflect the inherent ambiguity of the social (see Beauvoir, 1976).

## Practices of Missing: The (Absent) Other

Practices of missing cut to the core of Beauvoir’s farewell ceremonies: the (continued) relationship between mourner and deceased. In narrating the deaths of her mother and her partner Sartre, Beauvoir (1965, 1984) addresses the void in herself that marks their absence. In a similar way, the participants’ practices of missing make sense of the more-than-absence of the deceased other: their continued hold on the mourner’s life, manifested in experiences of grief, loss, loneliness, continuous attachment but also surprise and renewal. In the following two quotes, the aspect of loneliness is especially prominent:

Yes, and then I think: yes, well, then [a relationship] will no longer be possible for me. I think: geez, huh. […] And that’s the other perspective of course. Friends, a married couple growing old together, and of course we hoped that too. (Elske, 73)

Well, I’m on my talking chair now and I have something to talk about. But during the day I have nothing to talk about. You also can’t be on the phone constantly and all that stuff. Get me? That’s what I hand in. I’m inside and completely thrown back on myself. And that’s pretty damn hard. (Oved, 83)

Both Elske and Oved discuss the loneliness they experience after the deaths of their partners and friends. Elske mentions her hope to grow old together with her partner, which was tragically cut short by his premature death. She realises her loneliness through a practice of missing: comparing her life with the lives of other couples where both partners are still alive. Looking at them, she misses the possibility of a shared life, which, given her advanced age, seems lost for good. Oved’s feelings are similar. He suffers from loneliness and depression following the dissolution of much of his social circle. Oved misses his friends as he “sits on his talking chair” where the weight of his situation both subsides and continues to press down on him. Being in company, he is glad to have someone to talk to; at the same time, he is confronted with his loneliness during the rest of the day. Being thrown back on himself, Oved feels like a “pathetic old little man,” as he said at another point during the interview.

Other practices of missing focus less on one’s own loss and more on the quality of the continued relationship with the deceased. This is illustrated by the following two quotes:

But… yes… once again, why my mother went on such a rampage, I will never get an answer to that. She took that to the grave with her. And that made me very angry. (Hugo 69)

But occasionally she would do things that made me think: mum, you can’t do that! But I found that so annoying then. […] And, um, I remember when I saw her, when she had died. Yes, then suddenly I was like: uh… yes, then I looked at her so differently. Then I was so mild towards her again. I was no longer mad at her. (Eveline, 65)

When Hugo and Eveline describe their feelings toward their deceased mothers, they both highlight instances of anger. Eveline’s mother’s apparently groundless allegations against her caregivers put her daughter in an uncomfortable position. Any hard feelings that Eveline had harboured, however, dissipated after her mother’s death. This did not happen in the case of Hugo, who survived years of severe physical abuse at the hands of his mother. The object of his persisting anger is the lack of answers and of answerability. For Hugo, then, to miss his mother is to ask about the reasons for her violence—without ever getting an answer.

In contrast to active practices of loneliness and anger, there were also instances where participants felt overwhelmed by grief:

But, look… this means: I, I’m the only… one left. There’s nothing left [starts crying], nothing. (Oved, 83)

Because sometimes someone says nothing and that’s more helpful than all kinds of talk or all kinds of words. Words are sometimes a bit…… so. (Elske, 73)

In their own ways, Oved and Elske express grief and loneliness beyond words. Oved ends up crying as he enumerates the many losses he has endured in recent years. His conclusion cuts to the core of his loneliness: “I’m the only one left.” Elske makes a similar point and explicitly addresses the impotence of language. Her reflection on the uselessness of words of solace ends with a long pause, followed by an indefinable “so.” In these instances, Oved and Elske collapse in the absence of the deceased rather than relating to them in a practice of missing. Grief that remains unacknowledged or that does not fit into an established order of missing can have this destabilizing effect. Yet, it can also surprise the mourner and open up unexpected possibilities, as illustrated by another story of Arie’s. In the following quote, he talks about visiting his husband Daan at the hospice and about sharing the burden of care with Daan’s boyfriend Pablo:

But Pablo was there too. And then at some point I left and Pablo still stayed with him. I had no problem with that either. I thought: well, Daan, that’s just fine for Daan. Because what does he have to lose? What do I have to lose? He’s going to die anyway. So…… I’m happy for him. (Arie, 71)

The marriage of Arie and Daan was open, but not to the extent that they would meet each other’s boyfriends. Pablo’s active role in caring for Daan was, therefore, a novelty. Arie, on his part, was surprised by his lack of jealousy. An outsider might have considered them “crazy,” he noted at another point. Yet, Arie took no offence and generously made space for his husband’s boyfriend. With Daan’s death on the horizon, Arie engaged in a practice of anticipated missing: he learned to love him fearlessly, in ways that he had not expected. More event than practice, missing Daan became an occasion for reconfiguring their relationship and embracing a more expansive, “promiscuous” form of care (Chatzidakis et al., 2020).

Participants’ practices of missing address the more-than-absence of the deceased other and tend toward new meaning. This finding resonates with approaches in bereavement research that construe mourning as meaning reconstruction (Neimeyer, 2001) and highlight the “continuing bonds” between mourner and deceased (Klass et al., 1996). At the same time, practices of missing also capture moments of rupture and meaningless. Following the death of his closest friends, Oved did not manage to reconstruct the meaning of his life but spiralled into despair and loneliness. His situation is not unique and is hardly pathological; rather, it reflects the existential weight of bereavement overload and its unpredictable effects on the quality of later life (Kastenbaum, 1969; Moss & Moss, 2003). Besides the risks of rupture and meaninglessness, practices of missing also capture the positive excesses of grief: unexpected eruptions of new meanings and attachments. Arie’s erotic generosity at the end of his husband’s life is a case in point. Another interesting line of inquiry connects the reconstitution of meaning to the subject of materiality (see Lykke, 2022; Richardson, 2014). This connection is apparent in Eveline’s and Hugo’s ambiguous discourses on their mothers’ corpses, caught between living sense and dead matter.

## Discussion and Conclusion

The framework of the farewell ceremony offers a new perspective on ageing that reflects the existential situation of older people. As a person ages past midlife, they are not only confronted with the repeated deaths of loved ones; these losses also act as reminders of their own mortality. This interweaving of one’s own death and the other’s death has gone largely unnoticed in mainstream approaches to ageing, death, and bereavement, which tend to extrapolate from the experience of younger and middle-aged people. The suggestion is that people only ever face one loss at a time, whereas the accumulation of losses over the life course and the intersection of different types of losses are not taken into account. Building on Beauvoir’s philosophy of existence, this paper has explored different kinds of “farewell ceremonies”—practices of timing, communing, and missing that capture different ways in which older people make sense of the losses of later life. In the following, we will explore the concept of the farewell ceremony in more detail by looking at its constitutive parts: its performative power, its contextual character, its presumption of futurity or lack thereof, and its tendency to blur the boundary between self and other.

Practices of timing, communing, and missing are the very stuff of farewell ceremonies, ranging from the mundane (e.g., writing a text message) to the extraordinary (e.g., looking at the dead body of one’s mother). Like Beauvoir’s farewell ceremonies of writing, these practices are not *about* grief, that is to say, they do not represent or reflect on it. They rather *perform* grief, thereby making manifest the living unity between meaning and its expression (see Merleau-Ponty, 2010). This expressive unity of meaning and expression would sometimes emerge in the interview situation itself, when, in the face of grief, speech gave way to stammers, pauses, and bouts of tears. In a sense, farewell ceremonies can say more than words; they assume a situation not only verbally but also in its affective and embodied dimensions.

The existentialist conception of practice as the assumption (a way of making sense) of a situation resonates in certain respects with the conception of ageing as an accomplishment, a kind of “doing” (see Höppner & Wanka, 2021; Schroeter, 2005). According to Laz (1998, 2003), age is not a natural property of individuals but the result of everyday interactions between people and their social environments. To consider age as an accomplishment in this way directs attention to the micro-practices through which older people come to embody their age. The framework of the farewell ceremony puts similar emphasis on practices but is more specific on the context in which they operate. Timing, communing, and missing are not generic interactions between people and environments but carry specific affective meanings. The blending of grief and loneliness or the sense of “completing the circle” are examples of such meanings, rooted in the existential situation of later life. By tending to later life in its affective-existential specificity, the framework of the farewell ceremony can add nuance and sophistication to discussions of ageing as an accomplishment.

Where the two approaches differ, however, is in their conception of sense-making. Beauvoir’s philosophy of existence, from which the framework of the farewell ceremony is derived, assumes that sense-making is the work of a free subject. In her view, human existence is free by virtue of its capacity to transcend its present situation and to project itself toward a future goal (Vetter, 2004: 144). What used to be an impersonal given is thus transformed into the stuff of a personal life story, which is the precise meaning of sense-making. Participants’ farewell ceremonies demonstrate that, while loss is an inevitable fact of life, its meaning depends on the ways in which people assume it. This assumption of loss draws on a person’s situation and previous life choices (see Beauvoir, 1996: 441). It is not surprising, therefore, to find great diversity in participant’s farewell ceremonies.

Despite their diversity, practices of timing, communing, and missing share a presumption of futurity: the faith in a future beyond loss and death. A person expresses such faith as they invest in new projects or relationships following a confrontation with death. Where the presumption of futurity is lacking, the person may descend into sadness and despair. Existence no longer flies toward possibility and the person becomes trapped in a sterile present, closed off from its living relationship to the past. Beauvoir (1996: 366) describes how repeated bereavement erodes the living memory of the other, which is premised on their bodily presence, and chips away at subjectivity itself. We have seen this spiral of despair at work in the stories of participants, such as Evert, who described feeling surrounded by illness and death, and Oved, who suffered from severe sadness following the death of his closest friends. Their movement toward despair is neither a pathological condition nor the result of weak will; it is the expression of an existential situation in which loss has grown to overpowering proportions. The absence of possibility is a real possibility of existence in later life. It suggests an existential limit to healing and sense-making that does not receive sufficient attention in the literature on meaning reconstruction (Neimeyer, 2001), continuing bonds (Klass et al., 1996), and gerotranscendence (Tornstam, 2005).

The last component of the farewell ceremony that we want to discuss is the role of the other. Farewell ceremonies come with a vivid intersubjective horizon. They unfold in networks of relationships that include not only the deceased other but also supportive others, desired others, and the other within the self. To bid farewell to a loved one, to bury them, reorganises one’s intersubjective possibilities. As one form of togetherness becomes impossible, other possibilities come into view, such as the possibility of one’s own death or the possibility of other relationships. This blurring of the perspectives of self and other is characteristic of Beauvoir’s philosophy of death. Similarly, she dedicates her *Adieux* (Beauvoir, 1984) not to Sartre, who has ceased to exist, but to those who come after him and might carry forward his projects. The ceremonial aspect of farewell ceremonies consists in this opening toward others. Like more formalised rituals, farewell ceremonies lift an individual practice out of the present moment. They imply a larger social context in which meaning is personal but not private and where it forms part of an intersubjective project of becoming.

The blurring of the perspectives of self and other is a significant finding of this research. Frequently, practices of timing, communing, and missing blur the boundary between self and other, thus challenging the Heideggerian (1962) account of being towards death and its constitutive disregard for the death of the other. Our findings resonate with Beauvoir’s philosophical interpretation of death, according to which one’s own death, rather than being a beacon of authenticity, is really the death of another. Beauvoir understands death as an “unrealisable,” an external fact about a person that they cannot assume fully. As she states in *The Coming of Age*: “it is the Other who is mortal in my being. I know that I am mortal, just as I know that I am old, by adopting the outsider’s view on me” (Beauvoir, 1996: 441). In the Beauvoirian view, which we have developed in this paper, there is no radical difference between one’s own death and the death of a loved one—both are sense-less, external outrages against existence. By virtue of this common unrealisability, there is a line of correspondence between one’s own death and the death of the other. I can identify with a dead or dying other, precisely because my own death does not properly “belong to me”.

This paper started with the common-sensical assertion, voiced by our participant Arie, that older age inevitably comes with loss and bereavement: “that’s just the way it is, that’s just the way it *is*.” We have aimed to show that this apparently self-evidence of loss and bereavement is premised on diverse and ambiguous practices of sense-making. This diversity of practices reflects the diversity of positions from which participants assumed their situation. Shared personal characteristics, such as age cohort or gender, sometimes overlapped with shared attitudes and beliefs, though the small sample size did not allow us to draw strong links between the two. We have rather focused on participants’ individual differences (and commonalities) in assuming their situations. Older people need to assume the biological, social, and cultural givens of their situations in order for these givens to be experienced *as* something, to have meaning *as* something: a terrible loss, a reminder of finitude, a reason for gratitude, the will of God, an inevitability etc. We have elaborated on this classical existentialist position by highlighting the concrete practices that go into the making of sense, which we have conceptualised as “farewell ceremonies.”

The farewell ceremonies of timing, communing, and missing that we described in this paper are distinct ways of weaving together the facts and meanings of mortality in later life. They demonstrate that making sense of death and loss does not require deep metaphysical meditation but happens in the countless micro-practices people engage in every day. The sum of these practices sets older age apart from earlier life stages. As we have shown, the meanings of death and loss are not fixed but tend to change with age. What may appear shocking and outrageous to people of middle age—the death of family members, friends, and acquaintances—becomes increasingly expected as people grow older. This incremental shift in the meaning of death and loss is insufficiently captured by schematic distinctions, such as between the “young old” and the “old old.” Being expected, bereavement can still feel life-shattering, depending on the concrete context of the loss. Thus, while repeated bereavement may be traumatic, it may also yield a new understanding of one’s own mortality: not as the tragic fate of an individual but as a condition shared with others. Beauvoir (1996: 367) said it well when she noted that “[i]n the ‘monuments of the dead’ that stud my history, it is I who am buried.” Hers is a vision of a slow denouement of sense, a death by repeated bereavement. While her account appears to be bleak, it also invites us to re-think mortality from the point of view of older age: as a continuous farewell.

## Footnotes

1 There is a sizable body of literary scholarship investigating the psychological motivations (Idt, 1991; Marks, 1998; Moi, 1994) and literary strategies (Bjørsnøs, 2013; Fort, 2007; Tidd, 1999) underlying Beauvoir’s “writing against death” (Bainbrigge, 2005). Philosophical interpretations, by contrast, have explored Beauvoir’s conception of death in relation to femininity, freedom, and intersubjectivity (Heinämaa, 2010b; Holveck, 1989; La Caze, 2004–2005).

2 Participants were requested to pick a photo of themselves around twenty years ago. The photo served as an opening to a conversation on the passage of time and felt differences between the present and the past.
